# Personalized Simulation Modeling of Overlapping Microwave Ablation for Large Tumors

**DOI:** 10.3390/bioengineering13040421

**Published:** 2026-04-02

**Authors:** Qi Wang, Shuicai Wu, Luyu Li, Xinnan Xue, Honghai Zhang, Weiwei Wu, Hongjian Gao

**Affiliations:** 1 College of Chemistry and Life Science, Beijing University of Technology, Beijing 100124, China; 2 Beijing You’an Hospital, Capital Medical University, Beijing 100069, China; 3 College of Biomedical Engineering, Capital Medical University, Beijing 100069, China

**Keywords:** finite element simulation, overlapping microwave ablation, large tumor, coagulation zone, true anatomical structure, single-needle multi-point strategy

## Abstract

This paper evaluates the advantages of overlapping microwave ablation (OMWA) for the personalized treatment of large tumors, providing quantitative and technical references for conformal tumor eradication. A three-dimensional numerical model coupled with electromagnetic fields and Pennes’ biological heat transfer equation was constructed, comprehensively considering the nonlinear behavior of tissue electrical and thermal parameters with temperature changes. A simulation model was developed to predict temperature distribution and the formation of the coagulation zone under single-needle multiple-point and multiple-needle multiple-point OMWA strategies. The LiTS2017 public dataset of liver tumor cases and real clinical cases was selected for verification. The results showed that OMWA could achieve faster thermal accumulation, higher central temperature, and more conformal tumor coverage. Compared with the single-needle strategy, OMWA significantly reduces thermal damage to surrounding healthy tissues while achieving complete tumor coverage. Therefore, OMWA is more efficient and safer than the single-needle strategy in the personalized treatment of large tumors and can provide important references for clinical preoperative planning and parameter optimization.

## 1. Introduction

Primary liver cancer is the sixth most common cancer worldwide and the third leading cause of cancer death [1]. Surgical resection remains the main treatment option [2]. However, due to limitations such as tumor size, location, and complications, many patients do not meet the surgical criteria. For patients who cannot be resected, thermal ablation techniques have become an effective alternative [3]. Among them, radiofrequency ablation [4] (RFA) and microwave ablation [5] (MWA) are the most commonly used modalities. RFA generates heat energy through alternating current to achieve tissue coagulation and necrosis, but its efficacy is easily affected by carbonization and heat sinking [6,7]. In contrast, MWA generates heat by inducing the high-frequency oscillation of water molecules through electromagnetic waves, which effectively mitigates the heat-sink effect [8]. Compared to RFA, MWA achieves larger ablation volumes [9], higher tissue temperatures [10], more uniform necrosis, and shorter treatment times, while offering a superior local tumor control rate [11].

Image-guided MWA has shown higher conformal coverage efficiency in the treatment of large tumors [12,13]. The morphology and volume of the ablation zone are affected by tissue thermal conductivity, blood perfusion rate and dielectric properties, and these parameters vary significantly under different pathological conditions [14]. Therefore, establishing personalized simulation models is of great significance for preoperative planning.

The finite element method (FEM) has shown good performance in MWA simulation [15]. Radmilović-Radjenović [16] utilized COMSOL to construct a realistic liver model, analyzing the impact of various parameters on the ablation range. Bošković [17] pointed out that modeling based on real geometry can more accurately predict temperature distribution and tissue damage. Gupta [18] and Wu [19] further proved that by optimizing the antenna structure and input power, the conformality and accuracy of MWA can be improved. Heshmat [20] combined patient-specific models to verify the accuracy of predicting the ablation zone and the minimum ablation boundary.

Currently, most research on personalized MWA focuses on single-needle strategies, lacking systematic studies on large or irregular tumors. This study proposes an OMWA strategy based on real clinical ablation procedures to achieve conformal coverage of irregular and large tumors. The OMWA mainly includes two methods: multiple insertions of a single antenna to form an overlapping coagulation zone or simultaneous heating of multiple antennas to generate a fusion coagulation zone. This paper uses a realistic anatomical model constructed based on the LiTS2017 dataset for simulation and validates the results with clinical cases. This study can more accurately assess temperature distribution and tissue damage, providing a basis for preoperative planning and parameter selection for personalized ablation of large tumors.

## 2. Materials and Methods

### 2.1. Construction of Real Anatomical Models

This study selected three representative liver models from the LiTS2017 dataset (Liver Tumor Segmentation Challenge 2017), covering pathological features such as normal morphology and complex vascular structures [21]. At the same time, tumor cases of different sizes, locations and numbers were selected to verify the adaptability of the OMWA strategy in diverse clinical scenarios [22]. The liver and tumor features are shown in Table 1. In the modeling process, based on the voxel-level segmentation mask, three-dimensional morphological preprocessing was performed first, and then the Marching Cubes algorithm [23] was applied under the constraint of physical voxel spacing to extract isosurfaces. Taubin smoothing was combined to reduce jagged artifacts, and QEM mesh simplification was used to reduce computational complexity. Finally, the mesh models of the liver and tumor were reconstructed and stored in STL format for subsequent simulation and visualization.

**Figure 1:**
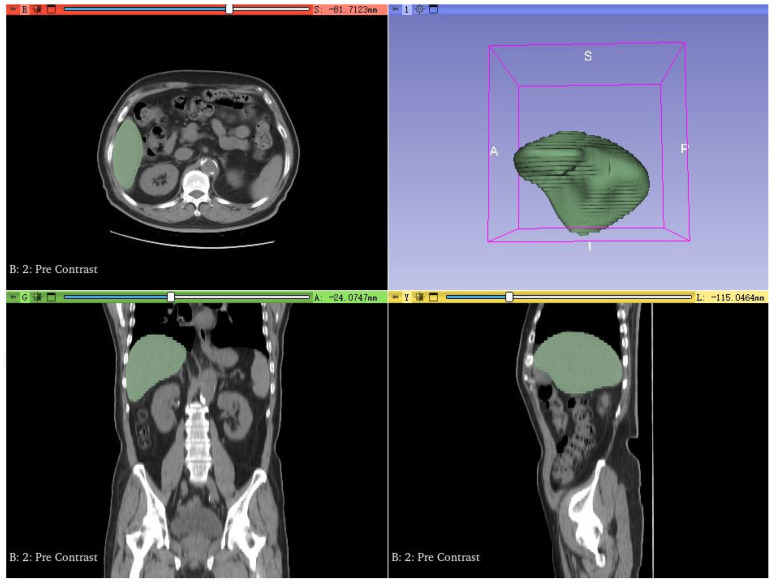
Schematic diagram of segmentation results for clinical patient liver CT images.

The clinical data in this study were obtained from enhanced CT scans of two patients with liver tumors at Beijing You’an Hospital, affiliated with Capital Medical University (Table 2). The study has been approved by the hospital’s ethics committee and informed consent has been obtained from the patients. All image data has been anonymized and complies with medical data protection regulations. The physicians first used version 5.4.0 of the 3D Slicer software [24] to complete CT image segmentation (Figure 1). Then they generated STL models of the liver and tumors through surface reconstruction algorithms for subsequent COMSOL simulation analysis.

### 2.2. Finite Element Simulation of OMWA

**Figure 2:**
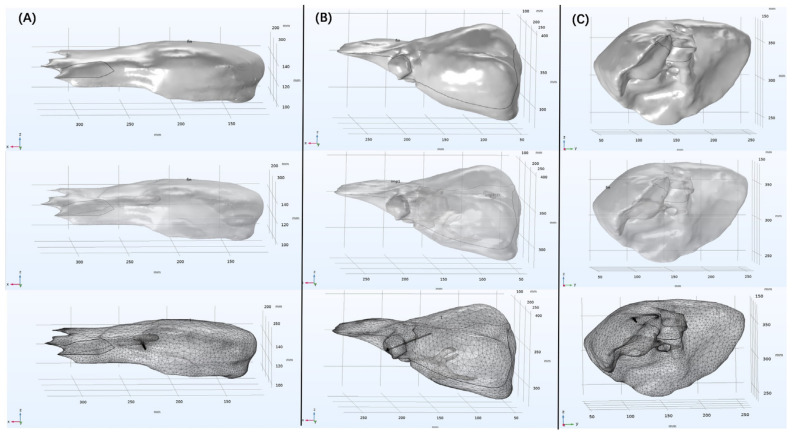
Schematic diagram of three-dimensional liver simulation models. Figures (**A**–**C**) respectively show the segmentation reconstruction results, COMSOL solid simulation model, and mesh generation results for patients 50, 88, and 90 from the LiTS2017 dataset. The numbers on each coordinate axis in the figure (unit: mm) represent the relative position and size range of the liver 3D reconstruction model in space.

**Figure 3:**
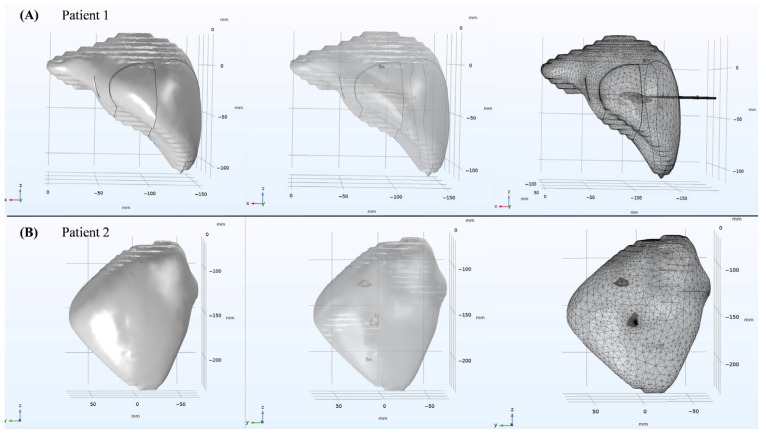
Schematic diagram of three-dimensional simulation models for clinical patients. Figures (**A**,**B**) respectively represent the segmentation reconstruction results, the COMSOL solid simulation model, and the mesh division results for clinical patient 1 and clinical patient 2.

In this study, the OMWA techniques were applied to a real liver anatomical structure to investigate the effects of different insertion positions and angles on the conformal coverage of large tumors. The STL file was imported into COMSOL Multiphysics software (v6.0, COMSOL Inc., Stockholm, Sweden) to create a three-dimensional simulation model, as shown in Figure 2 and Figure 3. The microwave antenna consists of an inner conductor, a dielectric, and a slotted outer conductor; its structural parameters are shown in Table 3. In this study, the microwave antenna used has a water-cooling circulation system [25]. This system maintains the temperature of the antenna shaft and suppresses the reverse heating effect. This ensures that microwave energy is concentrated at the radiation slot, forming a more regular and uniform ablation zone.

To simulate the clinical OMWA procedure, this study proposed a multi-target simulation model. The simulation process includes Study 1, Study 2, and Study 3. Study 1 corresponds to the first ablation and simulates the first ablation process of OMWA. Study 2 simulates the cooling and heat conduction process after the first ablation, reflecting the CT re-scan [26] and path planning stage after the first ablation in clinical practice. Study 3 corresponds to the second ablation and simulates the second ablation process of OMWA. This simulation technology can flexibly adjust the number of ablations and the path to achieve individualized treatment for different tumors, especially suitable for conformal coverage of large or irregular tumors [27].

The OMWA simulation model was solved by coupling the electromagnetic field and the biological thermal field. Electromagnetic energy deposition was calculated using the Helmholtz harmonic Equation (1) [28], and the specific absorptivity (SAR) in the tissue was calculated using Formula (2) [29]. The transient heat transfer in the tissue was solved using the Pennes classical biological heat transfer equation [30] (3). The relevant equations are as follows:

(1) $$\nabla \times \mu_{r}^{-1}(\nabla \times E)-k_{0}^{2}(\varepsilon_{r}-\frac{j\sigma }{\omega \varepsilon_{0}})E=0$$

(2) $$\rho \cdot SAR=\frac{1}{2}\sigma {\left|E\right|}^{2}$$

(3) $$\rho \cdot C_{p}\cdot \frac{\partial T}{\partial t}+\nabla (-k\nabla T)=\rho \cdot SAR+(T_{b}-T)c_{b}\omega_{b}\rho_{b}+Q_{met}$$

In the formula, $\mu_{r}$ is the relative magnetic permeability ($\mu_{r}$ = 1); E is the electric field strength (V/m); $k_{0}$ is the free space wavenumber; $\varepsilon_{0}$ is the relative permittivity of vacuum; $\varepsilon_{r}$ is the relative permittivity of biological tissue; σ is the electrical conductivity of biological tissue (s/m); ρ is the tissue density (kg/m^3^); *SAR* is the specific absorption rate (W/kg); $C_{p}$ is the specific heat capacity of the liver; $c_{b}$ is the blood’s specific heat capacity (J/(kg·℃)); $\omega_{b}$ is the blood perfusion rate (kg/(m^3^·s)); $Q_{met}$ is the heat generated by tissue metabolism (W/m^3^). The subscript “b” indicates parameters related to blood.

For clarity, all electrical and thermal parameters involved in the electromagnetic and bioheat models, together with their units and corresponding expressions or values, are summarized in Table 4. Table 5 shows the electrical and thermal parameters used in the tumor tissue [20].

During the OMWA process, the thermal and electrical parameters of liver tissue change with increasing temperature. To describe the real changes in these parameters, this study used temperature-related electrical parameters, a specific heat capacity function based on water content changes, and a linearly changing thermal conductivity function [33].

The specific heat capacity function based on the change in water content is [34]:

(4) $$C_{p}(T)=\left\{\begin{array}{cc} C_{p25{}^{\circ}C}+k_{w}\cdot (T-25) & T\leq 70\;{}^{\circ}C\\ C_{p70{}^{\circ}C}-\frac{\alpha }{\rho }\cdot \frac{\partial W(T)}{\partial T} & T>70\;{}^{\circ}C \end{array}\right.$$

(5) $$W(T)=\left\{\begin{array}{ccc} 0.778\cdot (1-\exp(\frac{T-106}{3.420})) & 70\;{}^{\circ}C\leq T<100\;{}^{\circ}C & \\ 7.053-0.0640\cdot T & 100\;{}^{\circ}C\leq T<104\;{}^{\circ}C & \\ 0.778\cdot \exp(-\frac{T-80}{34.370}) & 104\;{}^{\circ}C\leq T & \end{array}\right.$$

In the formula, $C_{P25{}^{\circ}C}$ and $C_{P70{}^{\circ}C}$ are the specific heat capacities at 25 °C and 70 °C, respectively; $k_{w}$ represents the temperature coefficient (J/(kg ${{}^{\circ}C}^{2}$)); α is the latent heat constant (set as 2260 kJ/kg); W(T) is a function of water content.

The expressions for the relative permittivity ε, electrical conductivity s (s/m), and thermal conductivity k(W/(m·°C)) are [33,34]:

(6) $$\varepsilon (T)=45\cdot \left[1-\frac{1}{1+\exp(5.200-0.0519\cdot T)}\right]$$

(7) $$\sigma (T)=2.2\left[1-\frac{1}{1+\exp(5.324-0.0607\cdot T)}\right]$$

(8) $$k(T)=k_{25{}^{\circ}C}+0.00111\cdot (T-25)$$

In the formula, $k_{25{}^{\circ}C}$ is the thermal conductivity at 25 °C.

### 2.3. OMWA Strategy

**Figure 4:**
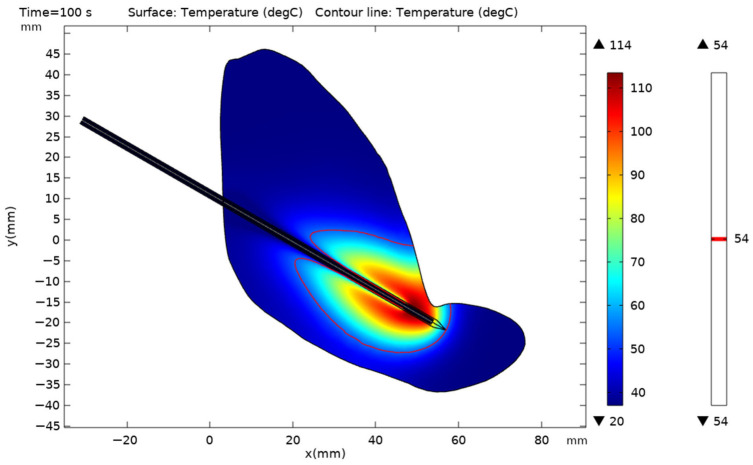
Schematic diagram of the threshold coagulation zone at the 54 °C isotherm.

The initial temperature was set to 37 °C to simulate the physiological baseline for in vivo liver ablation [35]. The water-cooled circulation device temperature is set to 20 °C. The model was meshed into free tetrahedral meshes using an automatic mesh generation method. The computational domain boundary was assigned ideal electromagnetic scattering conditions, while the outer surface of the liver model was specified as an adiabatic boundary. Denaturation will occur quickly when liver tissue is between 50 °C and 60 °C [34]. To use a unified standard to assess the coagulation zone, we adopted 54 °C as the necrosis threshold, which was used by previous scholars. This value was often used in the literature to define the boundary of irreversible thermal damage [36,37] (Figure 4).

To ensure a consistent measurement standard, this study implemented different ablation strategies by adjusting the action time and needle insertion angle under the same ablation power conditions. The execution of each strategy strictly followed the hard constraints of surgical navigation [38,39], while also taking into account the optimization objectives of soft constraints [40]. The OMWA strategies for different cases are shown in Table 6, with an ablation power of 50 W. In the table, “first ablation, second ablation, third ablation, and fourth ablation” represent the ablation time; surgical planning represents the time spent simulating clinical surgical path planning. Each case is tailored to a personalized ablation plan based on the tumor’s morphological characteristics. During planning, we prioritize minimally invasive strategies (single-needle ablation and single-needle multi-point ablation) whenever sufficient conformal coverage can be achieved. If these strategies fail to provide adequate tumor coverage or ensure sufficient safety margins, we use a multi-needle strategy. After each ablation, surgical planning is re-performed to assess conformal coverage. This is to determine if further ablation is needed. Larger tumors typically require two or more ablation procedures to achieve conformal ablation. Under the same case conditions, the total time to achieve conformal tumor coverage using the single-needle ablation strategy was 210 s, 8400 s, 2520 s, 130 s, and 210 s, respectively.

This study calculated and visualized the volume and morphology of the final coagulation zone, and plotted the coagulation zone within the tumor, at the tumor margin, and in surrounding healthy tissue. To evaluate the ablation effect, the following metrics used to quantify the relative damage degree of healthy tissue during ablation were defined: Ablation Margin (AM), the distance the ablation zone extends beyond the tumor boundary; Healthy Tissue Damage Volume (HTTDV), the volume of healthy tissue contained within the coagulation zone; and Healthy Tissue Damage Ratio (HTDR), the ratio of healthy tissue damage volume to the total ablation volume (HTDR = HTDV/AV). Finally, the OMWA strategy was compared with a single-needle ablation strategy that also achieved conformal ablation to assess the effectiveness and safety of different approaches. All simulation parameters and strategies were set under the guidance of clinicians.

### 2.4. Clinical Data and Validation Method

To verify the clinical feasibility of the proposed OMWA technique, this study implemented the overlapping ablation strategy on real patient data. By quantitatively analyzing and comparing preoperative, intraoperative, and postoperative CT images [32], the potential value of this technique in clinical application was systematically evaluated.

## 3. Results

### 3.1. Comparative Simulation Results of Ablation Strategies

**Figure 5:**
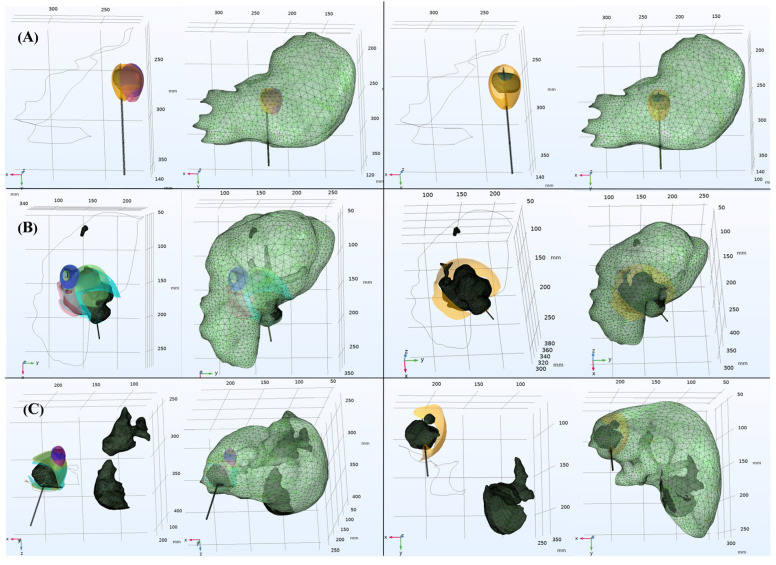
Three-dimensional coagulation zones formed under different ablation strategies in the LiTS2017 case. Figures (**A**–**C**) show the three-dimensional coagulation zones formed in patients 50, 88, and 90 of the LiTS2017 dataset, respectively, when implementing overlapping MWA and single-needle ablation strategies. Dark green grids indicate tumors, light green grids represent liver tissue, and differently colored ellipses denote coagulation zones formed by varying ablation frequencies. Yellow ellipses indicate coagulation zones generated by the single-needle approach. In the overlapping MWA strategy, the differently colored ellipses (e.g., blue, green, and orange) represent the size of the coagulation zone formed by each ablation, while the combined overlapping region illustrates the cumulative ablation coverage achieved by the multi-needle strategy.

Figure 5 compares the 3D coagulation zones obtained with OMWA (left column) and single-needle MWA (right column) under the same ablation power (50 W) for Cases 50, 88, and 90.

Case 50 (Figure 5A) represents a relatively regular tumor smaller than normal. OMWA achieved conformal coverage through overlapping dual-target ablation, while single-needle single-point ablation failed to completely cover the lesion under the same parameter settings. This indicates that a longer ablation time is required to achieve complete coverage. 

Case 88 (Figure 5B) involves a large, irregular tumor near the hepatic artery, which makes it more difficult to achieve conformal coverage with a single-point strategy. OMWA employed a multi-target overlapping workflow (four ablations) and expanded coverage by controlling pullback/deflection while adhering to the soft and hard constraints of path planning. The first two were performed using a single-needle multi-point overlapping method (pulling back and deflecting to the left). The last two were also performed using a single-needle pullback and deflection to the right. This approach effectively expanded the coagulation range without increasing the power. Single-needle ablation significantly increased the ablation time.

Case 90 (Figure 5C) comprised multiple irregular tumors and underwent three ablation procedures. The ablation path was replanned via CT after the first ablation, and the subsequent two ablation procedures achieved multi-target ablation through single-needle retraction and angle adjustments. Overall, OMWA improved tumor coverage efficiency at the same power level while avoiding unnecessary expansion of the coagulation area.

**Figure 6:**
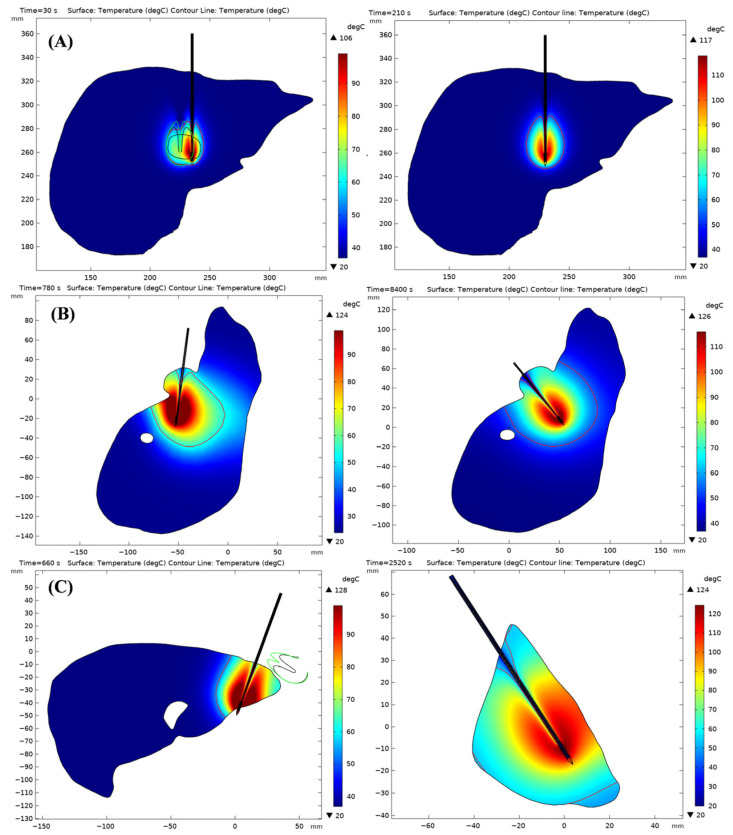
Temperature distribution results generated by the LiTS2017 case under different ablation strategies. Figures (**A**–**C**), respectively, depict the two-dimensional coagulation zones formed in patients 50, 88, and 90 from the LiTS2017 dataset when implementing OMWA strategies and single-needle ablation strategies. The colored vertical bars indicate temperature distribution. 54 °C represents the critical temperature for achieving complete tumor necrosis within the coagulation region.

Figure 6 visualizes the final temperature distribution for Cases 50, 88, and 90 under different ablation strategies. The 54 °C isotherm is used to delineate the thermal coagulation zone.

Case 50 (Figure 6A) showed a slightly smaller coagulation zone formed using OMWA. However, it better conformed to the tumor’s morphology, achieving conformal coverage of the lesion. This indicates improved clinical applicability of the technique.

Case 88 (Figure 6B) used OMWA to concentrate heating on the target area, resulting in a uniform temperature distribution. Heat was more concentrated in the tumor area, avoiding excessive heat diffusion. Simultaneously, it better protects surrounding normal liver tissue, reducing the risk of damage.

Case 90 (Figure 6C) had a long protrusion at the tumor edge. We chose to use two ablation needles for surgical treatment. First, the protruding tumor area was ablated, with a relatively short ablation time. Then, the main body of the tumor was ablated, requiring a longer ablation time. This better protected the surrounding normal liver tissue while achieving precise conformal coverage of the tumor.

In summary, these cases demonstrate that, at the same power settings, OMWA exhibits better conformal coverage of the tumor and less damage to the surrounding healthy tissue. This provides strong support for the precision treatment of complex tumors.

Table 7 reports the long-axis and short-axis diameters of the tumor and the final coagulation zone. In these three cases, OMWA generated smaller coagulation zones than single-needle ablation in both axes. Specifically, the long-axis diameter was reduced by 19.2% (Case 50), 6.2% (Case 88), and 22.7% (Case 90), while the short-axis diameter was reduced by 1.7%, 5.2%, and 12.9%, respectively. Moreover, the axis-wise excess beyond tumor dimensions was consistently smaller with OMWA (mean long-axis excess: 5.31 mm vs. 14.23 mm; mean short-axis excess: 5.68 mm vs. 9.20 mm). This indicates that OMWA causes less damage to healthy tissues while achieving conformal coverage of the tumor.

### 3.2. Clinical Validation

In practice, most interventional surgical cases are treated using the OMWA strategy. This study utilizes patient-specific modeling to formally describe and reproduce the clinical OMWA workflow for preoperative planning demonstrations. Simulation parameters (such as power and time) are set to closely approximate clinical reality; therefore, the simulated coagulation area is used only as a preoperative planning reference, not as a precise reconstruction of the actual intraoperative environment.

#### 3.2.1. Comparison of Results Between Overlapping Ablation and Single-Needle Ablation

**Figure 7:**
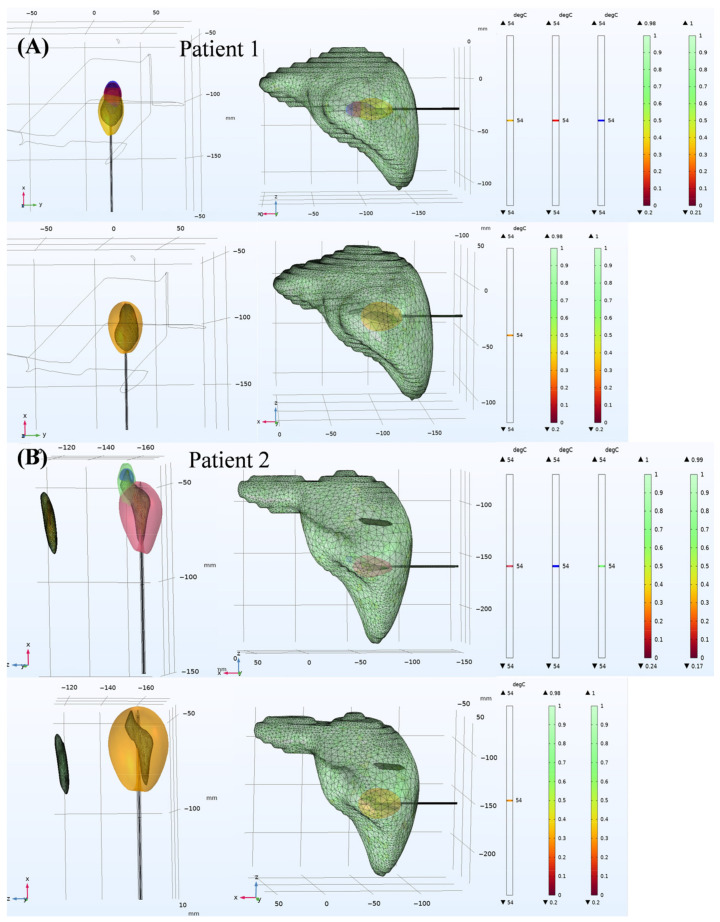
Comparison of three-dimensional coagulation zones between overlapping and single-needle MWA in clinical patients. Three-dimensional coagulation zones in clinical data. Figures (**A**,**B**) show the sizes of the three-dimensional coagulation zones (indicated by the 54 °C isotherm) for OMWA and single-needle ablation in patient 1 and patient 2, respectively.

Figure 7 compares the coagulation zones of patient 1 (Figure 7A) and patient 2 (Figure 7B) under single-needle ablation and OMWA strategies.

Patient 1 had a single, regular tumor (narrow anteriorly and wide posteriorly) in the liver. This patient underwent a single-needle multi-point ablation strategy, with a shorter first ablation time and a longer second ablation time. The results showed that the coagulation zone generated while achieving complete tumor coverage was slightly smaller than that of the single-needle ablation strategy.

Patient 2 had multiple and irregular tumors. While the long-axis values were similar (42.63 mm vs. 45.70 mm), the single-needle produced a notably larger short-axis extent than OMWA (32.15 mm vs. 21.22 mm). This indicates that in complex geometric structures, the single-needle ablation strategy causes greater damage to surrounding healthy tissues. The above results indicate that the OMWA strategy provides a treatment plan for the clinical management of large and complex tumors.

**Figure 8:**
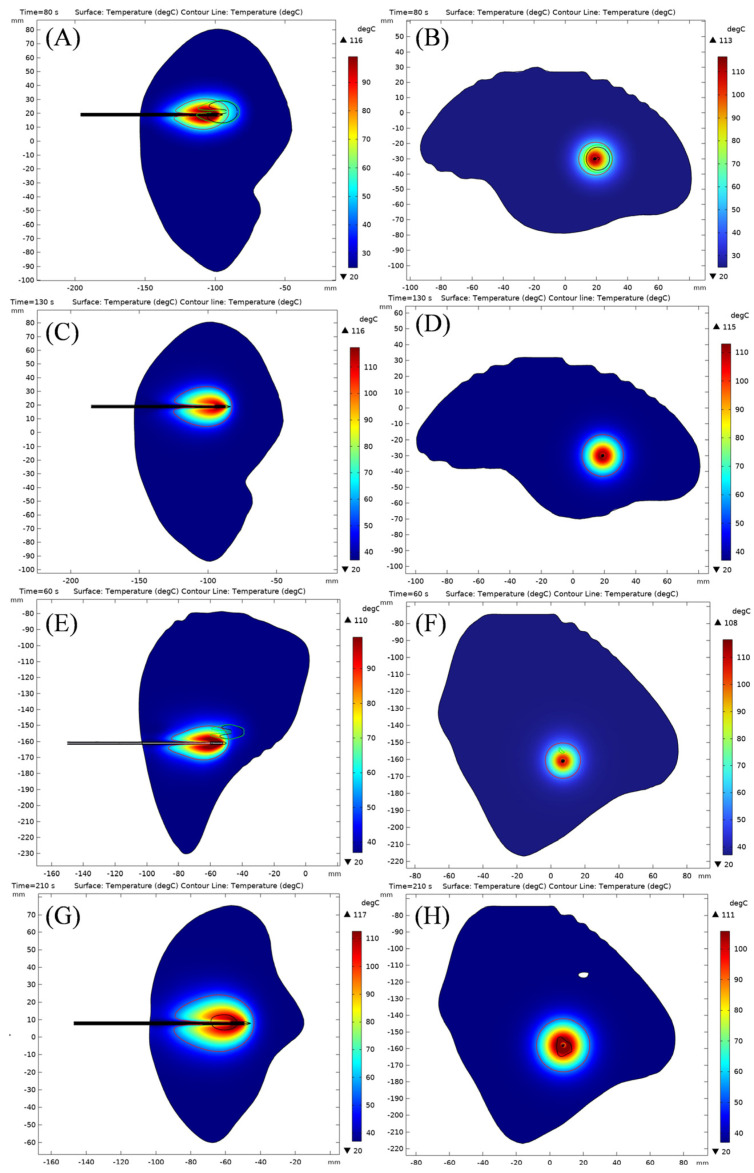
Two-dimensional coagulation zones and temperature distribution in patients 1 and 2 after single-needle ablation and OMWA. Figures (**A**–**D**) represent patient 1, and Figures (**E**–**G**) represent patient 2. Figures (**A**,**B**,**E**,**F**) represent the results of OMWA treatment. Figures (**C**,**D**,**G**,**H**) represent the results of single-needle treatment. The color bar represents the temperature gradient (°C). 54°C represents the temperature at which tissue reaches necrosis. The 54 °C curve in the figure represents the range of ablation necrosis at each stage.

Figure 8 illustrates the two-dimensional coagulation regions formed by single-needle ablation and OMWA in patients 1 and 2. In patient 1 (Figure 8A–D), the coagulation region formed by OMWA exhibits a slightly irregular shape, closely conforming to the tumor boundary. Single-needle ablation, aiming for conformal tumor coverage, produces a larger and more rounded coagulation region.

In patient 2 (Figure 8E–G), single-needle ablation, using a longer ablation time, resulted in a larger coagulation region, causing significant thermal damage to surrounding healthy tissue. In contrast, OMWA achieved precise, conformal tumor coverage with a significantly smaller coagulation region. These visual results demonstrate that OMWA achieves conformal tumor coverage while minimizing unnecessary thermal damage to surrounding healthy tissue.

Table 8 reports the long-axis and short-axis diameters of the tumor and the final coagulation zone in patients 1 and 2. In both patients, OMWA generated smaller coagulation zones than single-needle ablation in both axes. Specifically, in patient 1, the long-axis diameter reduced by 0.65%, while the short-axis diameter reduced by 21.5%. In patient 2, the long-axis diameter reduced by 6.7%, while the short-axis diameter reduced by 34.0%. Moreover, the axis-wise excess beyond tumor dimensions was consistently smaller with OMWA: in patient 1, the long-axis excess was 2.91 mm (OMWA) vs. 3.18 mm (single-needle), and the short-axis excess was 3.40 mm vs. 9.28 mm; in patient 2, the long-axis excess was 3.72 mm vs. 6.79 mm, and the short-axis excess was 4.90 mm vs. 15.83 mm. This indicates that OMWA causes less damage to healthy tissues while achieving conformal coverage of the tumor.

#### 3.2.2. CT Image Analysis

**Figure 9:**
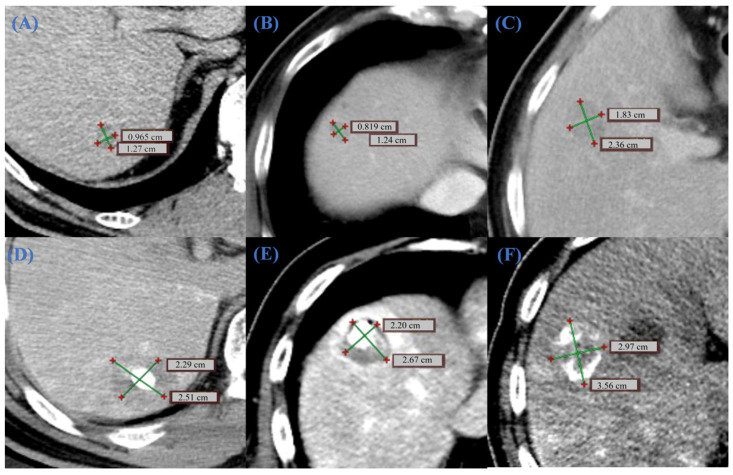
Comparison of tumor CT images before and after clinical surgical treatment. (**A**–**C**) shows the preoperative tumor size. (**A**) represents the CT image of patient 1, and (**B**,**C**) represent the CT images of different lesions in patient 2. (**D**–**F**) correspond to the immediate postoperative images at the aforementioned locations. The crosshairs in the images mark the long-axis and short-axis (cm) of the lesion and the postoperative coagulation zone. The comparison shows that the postoperative coagulation necrosis zone significantly exceeds the original tumor boundary, and both its long and short axis measurements are greater than the preoperative tumor morphology, confirming that the coagulation zone completely and effectively covered the target tumor. The green crosshairs indicate the measured axes of the lesion or coagulation zone, and the red markers denote the endpoints of the measurements.

In clinical surgery, CT scans play a crucial role in determining the ablation needle insertion point and in achieving surgical navigation [41]. To ensure a rigorous evaluation, the superiority of the OMWA strategy was assessed by comparing simulated OMWA outcomes against simulated single-needle outcomes under identical anatomical and physiological constraints. Retrospective clinical data (Figure 9) were presented independently to illustrate the clinical implementation of the OMWA strategy. To verify the clinical applicability of the proposed simulation method, Figure 9 illustrates the clinical effects before and after treatment in two representative patients. Patient 1 had a preoperative tumor length of 1.27 cm (Figure 9A). After ablation, the coagulated necrotic area expanded to 2.51 cm (Figure 9D), and imaging confirmed that the coagulated area completely covered the tumor. For patient 2, whose tumor morphology was more complex, multi-slice CT observation showed preoperative tumor diameters of 1.24 cm and 2.36 cm (Figure 9B,C). Immediate follow-up images after MWA (Figure 9E,F) showed significant morphological changes in the tumor area. The coagulated area measurements reached 2.67 cm and 3.56 cm, respectively. Quantitative analysis indicated that the postoperative ablation area was significantly larger than the original tumor volume, successfully establishing an ideal safety margin and ensuring effective local tumor control.

### 3.3. Evaluation Indicators

The quantitative indicators presented in Table 9 further confirm the clinical advantages of the OMWA strategy. These include ablation volume (AV), long and short axes of the safety margin, healthy tissue damage volume (HTDV), and healthy tissue damage ratio (HTDR). Compared to traditional single-needle ablation, OMWA provides more precise control over the safe zone. For example, in case 50, OMWA achieved a uniform safe zone (8.79 mm × 10.31 mm), while single-needle ablation resulted in over-ablation (17.25 mm × 10.84 mm).

A key quantitative finding is the significant reduction in healthy tissue damage volume (HTDV) and healthy tissue damage ratio (HTDR). In patient 2, OMWA reduced the HTDR to 0.66, while single-needle ablation reduced it to 0.88, effectively protecting surrounding healthy tissues while providing conformal tumor coverage.

Overall, these quantitative results provide a crucial reference for clinical decision-making. The OMWA strategy achieves a more uniform coagulation range at lower power settings, maximizing tumor destruction while minimizing damage to healthy tissues, thereby reducing the risk of thermal damage to adjacent critical structures. This provides a measurable, evidence-based reference for implementing overlap strategies in complex clinical MWA procedures.

## 4. Discussion

MWA is widely used as a minimally invasive treatment for liver tumors, and OMWA strategies for larger or irregular tumors have attracted increasing attention [42,43]. However, systematic simulation studies of OMWA surgical planning remain limited. Therefore, this study developed an OMWA simulation framework based on patient-specific anatomy to assist in preoperative planning and strategy design.

The innovation of this study lies in the simulation modeling of OMWA based on real anatomical structures, and the comparison and analysis of its ablation effect with single-needle ablation under the same power conditions. Large-volume tumors were specifically selected as the research object. Initially, a single-needle multi-point ablation strategy was used. When the tumor area was large and ideal coagulation coverage could not be achieved, a multi-target overlapping ablation strategy was further adopted to achieve personalized conformal treatment. This study used the publicly available LiTS2017 dataset and clinical cases for validation. The results show that OMWA can achieve sufficient tumor coverage in a short time and effectively reduce thermal damage to surrounding healthy tissues. The results of this study provide reliable quantitative evidence for clinicians in preoperative planning and treatment parameter optimization.

Despite achieving relatively ideal validation results, this study still has certain limitations. Firstly, the same ablation power parameters were used in all experiments to maintain consistency. However, in clinical practice, doctors typically adjust the power flexibly based on individual patient tolerance and lesion location. Furthermore, the current simulation assumes a static anatomical environment and does not account for tissue deformation caused by needle insertion. The liver is a viscoelastic organ, and interaction with the microwave antenna can lead to local tissue displacement and compression. While our results demonstrate that the 54 °C isotherm can approximate the necrotic region, neglecting these displacements is a limitation. Although the water function parameters we used can approximate the temperature distribution, future models will focus on a fully coupled porosimeter and biomechanical framework to account for density changes, evaporation, and tissue deformation during the procedure.

It should be noted that the superiority of OMWA was derived by comparing the results of OMWA with those of single-needle MWA under the same conditions. Although this idealized environment simplifies complex factors such as respiratory movements and needle deflection, it provides an important quantitative reference for preoperative planning. This will further improve the possibility of personalized ablation planning in highly deformable anatomical regions. Moreover, this study has not considered the blood perfusion effect. Blood flow, as an important heat-sink factor, significantly affects local temperature distribution and the formation of the coagulation boundary. This study did not reconstruct or simulate complex tissue structures such as bone, skin, and blood vessels, which have potential impacts on energy conduction and heat distribution during actual ablation. Finally, while this study provides preliminary validation of the OMWA strategy through a combination of numerical simulation and typical clinical case analysis, certain limitations remain. the limited number of clinical samples in this study may affect the method’s generalizability and applicability, and current work primarily focuses on the feasibility and individualized precision of the OMWA strategy. The inclusion of retrospective clinical cases aimed to demonstrate the existence of OMWA in actual clinical surgery. Although the clinical sample size in this study was limited, the proposed personalized modeling method can still provide quantitative decision support for clinicians, compensating for the problem of over-reliance on physician experience in clinical diagnosis and treatment. Future research plans to conduct larger-scale, multi-center clinical trials to further validate the practicality and long-term efficacy of the proposed model in a broader patient population. In addition, differences in ablation power, blood perfusion, and multi-tissue reconstruction should be considered to improve the clinical applicability and planning accuracy of the proposed framework and provide a reference for clinicians in preoperative surgical planning.

## 5. Conclusions

This study established a patient-specific OMWA simulation framework based on real anatomical structures and systematically compared it with a simulation-based single-needle reference under the same power conditions. The results showed that OMWA can achieve conformal coverage of large tumors expeditiously. Simultaneously, it effectively reduces thermal damage to surrounding healthy tissues. The multi-target OMWA simulation method can provide clinicians with quantitative references for preoperative planning and parameter optimization. Importantly, the current framework is intended for planning and optimization rather than for clinical outcome prediction (e.g., local control, recurrence, complications, or survival), and further prospective validation is warranted.

## Figures and Tables

**Table 1:** Physical characteristics of some cases from the LiTS2017 dataset.

Tissue	Liver-50	Tumor-50	Liver-88	Tumor-88	Liver-90	Tumor-90
Volume (mm^3^)	713,348.00	3077.00	1,558,027.20	116,660.55	1,717,439.97	123,061.00
Number	1	1	1	2	1	3
Shape	Flat and long	Regular shape	Normal	Giant multiple	Normal	Multiple irregular

Note: 50, 88, and 90 represent the patient numbers 50, 88, and 90 in the LiTS2017 dataset.

**Table 2:** Physical characteristics of some cases from clinical data.

Tissue	Liver-1	Tumor-1	Liver-2	Tumor-2
Volume (mm^3^)	954,980.00	2983.76	1,238,650.00	2873.64
Number	1	1	1	2
Shape	Slightly smaller	Irregular	Normal	Multiple and irregular

**Table 3:** Dimensions of the 2450 MHz microwave antenna.

Material	Dimension (mm)
Antenna outer diameter	1.9
Antenna inner diameter	1.3
Distance from the transmitting point to the antenna tip	11.5
Distance from the transmitting point to the antenna bottom	90
Total length	101.5

**Table 4:** Tissue electrical and thermal parameters used in the model.

Parameters	Description	Units	Corresponding Formula
$C_{p}$(T)	Specific heat capacity	J/(kg·°C)	Equations (4) and (5)
W(T)	Water content	Dimensionless	Equation (5)
ρ	Tissue density	kg/m^3^	ρ=1080
$\omega_{b}$	Blood perfusion rate	1/s	$\omega_{b}$ = 0.0004 [31]
$c_{b}$	Specific heat capacity of blood	J/(kg·°C)	$c_{b}$ = 3600
$T_{b}$	Initial tissue temperature	°C	$T_{b}$ = 37°C
$Q_{met}$	Metabolism	W/m^3^	$Q_{met}$ = 33,800 [32]
ε(T)	Relative permittivity	Dimensionless	Equation (6)
σ(T)	Electrical conductivity	S/m	Equation (7)
k(T)	Thermal conductivity	W/(m·°C)	Equation (8)
$\mu_{r}$	Relative magnetic permeability	Dimensionless	$\mu_{r}$ = 1
$k_{0}$	Free space wavenumber	1/m	$k_{0}$ = 2πf/c
$\varepsilon_{0}$	Permittivity of vacuum	F/m	$\varepsilon_{0}$ = 8.854$\;\times 10^{-12}$

Note: The subscript “b” denotes blood-related parameters. Temperature-dependent parameters are defined by Equations (4)–(8).

**Table 5:** Tissue parameters of tumors.

Tissue	$C_{p}$ (J/(kg·°C))	ρ (kg/m^3^)	ε(T)	σ(T) (S/m)	k(T) (W/(m·°C))
Tumor	3960	1040	Equation (6)	Equation (7)	Equation (8)

**Table 6:** OMWA strategy for different cases.

Ablation Strategy	Case 50 (s)	Case 88 (s)	Case 90 (s)	Patient 1 (s)	Patient 2 (s)
First ablation	80	180	50	30	10
Surgical planning	40	40	40	40	10
Second ablation	30	1020	660	70	60
Surgical planning	*	40	40	*	*
Third ablation	*	1200	10	*	*
Surgical planning	*	40	*	*	*
Fourth ablation	*	920	*	*	*
Total time	150	3400	800	140	80

Note: * indicates that this ablation or surgical procedure was not planned in this case. Steps are listed in chronological order. “Surgical planning” denotes intra-procedural path planning time after each ablation to reassess coverage.

**Table 7:** Morphological characteristics of the coagulation zone in LiTS2017.

Morphological Characteristics		Long-Axis (mm)	Short-Axis (mm)
Case 50	Overlapping	35.58	31.04
	Single needle	44.04	31.57
	Tumor	26.79	20.73
Case 88	Overlapping	75.76	71.35
	Single needle	80.78	75.25
	Tumor	72.76	68.66
Case 90	Overlapping	45.06	41.64
	Single needle	58.32	47.78
	Tumor	40.91	37.61

**Table 8:** Clinical data: two-dimensional coagulation zone size.

Morphological Characteristics		Long-Axis (mm)	Short-Axis (mm)
Patient1	Overlapping	41.28	21.75
	Single needle	41.55	27.63
	Tumor	38.37	18.35
Patient2	Overlapping	42.63	21.22
	Single needle	45.70	32.15
	Tumor	38.91	16.32

**Table 9:** Comparison of simulated ablation performance indicators between OMWA and single-needle strategies.

Case		Safety Margin (mm)		Ablation Volume (mm^3^)	HTDV (mm^3^)	HTDR (mm)	Tumor Volume (mm^3^)
		Long Axis	Short Axis				
Case 50	Overlapping	8.79	10.31	11,941	8864	0.74	3077.00
	Single needle	17.25	10.84	24,582	21,505	0.87	3077.00
Case 88	Overlapping	3.00	2.69	169,316	52,655.45	0.31	116,660.55
	Single needle	10.16	6.59	242,558	125,897.45	0.52	116,660.55
Case 90	Overlapping	4.15	4.03	49,563	8542.67	0.17	41,020.33
	Single needle	17.41	10.17	81,770	40,749.67	0.50	41,020.33
Patient 1	Overlapping	3.91	3.40	9861.9	6878.14	0.70	2983.76
	Single needle	3.18	9.28	16,215	13,231.24	0.82	2983.76
Patient 2	Overlapping	3.72	4.90	8399	5525.36	0.66	2873.64
	Single needle	6.79	15.83	23,040	20,166.36	0.88	2873.64

Note: All data in the table are derived from numerical simulation results of patient-personalized models.

## Data Availability

Data and materials will be made available on request.
